# Mixed Oxide Layered Double Hydroxide Materials: Synthesis, Characterization and Efficient Application for Mn^2+^ Removal from Synthetic Wastewater

**DOI:** 10.3390/ma13184089

**Published:** 2020-09-15

**Authors:** Cristina Modrogan, Simona Cǎprǎrescu, Annette Madelene Dǎncilǎ, Oanamari Daniela Orbuleț, Eugeniu Vasile, Violeta Purcar

**Affiliations:** 1 Analytical Chemistry and Environmental Engineering Department, Faculty of Applied Chemistry and Materials Science, University Politehnica of Bucharest, 1–7 Polizu Str., 011061 Bucharest, Romania; cristina.modrogan@upb.ro (C.M.); oanamari.orbulet@upb.ro (O.D.O.); 2 Inorganic Chemistry, Physical Chemistry and Electrochemistry Department, Faculty of Applied Chemistry and Materials Science, University Politehnica of Bucharest, 1–7 Polizu Str., 011061 Bucharest, Romania; 3 Institute of Research and Development “Metav” SA, C.A. Rosetti 31, 020011 Bucharest, Romania; eugeniu.vasile@upb.ro; 4 The National Institute for Research & Development in Chemistry and Petrochemistry—ICECHIM, Splaiul Independentei No. 202, 060021 Bucharest, Romania; violeta.purcar@icechim.ro

**Keywords:** mixed oxide LDHs, manganese, wastewater, adsorption kinetics

## Abstract

Magnesium–aluminum (Mg-Al) and magnesium–aluminum–nickel (Mg-Al-Ni) layered double hydroxides (LDHs) were synthesized by the co-precipitation method. The adsorption process of Mn^2+^ from synthetic wastewater was investigated. Formation of the layered double hydroxides and adsorption of Mn^2+^ on both Mg-Al and Mg-Ni-Al LDHs were observed by X-ray diffraction (XRD), Scanning Electron Microscopy (SEM) and Energy Dispersive Spectrometry (EDX) analysis. XRD patterns for prepared LDHs presented sharp and symmetrical peaks. SEM studies revealed that Mg-Al LDH and Mg-Al-Ni LDH exhibit a non-porous structure. EDX analysis showed that the prepared LDHs present uniformly spread elements. The adsorption equilibrium on these LDHs was investigated at different experimental conditions such as: Shaking time, initial Mn^2+^ concentration, and temperatures (10 and 20 °C). The parameters were controlled and optimized to remove the Mn^2+^ from synthetic wastewater. Adsorption isotherms of Mn^2+^ were fitted by Langmuir and Freundlich models. The obtained results indicated that the isotherm data fitted better into the Freundlich model than the Langmuir model. Adsorption capacity of Mn^2+^ gradually increased with temperature. The Langmuir constant (K_L_) value of Mg-Al LDH (0.9529 ± 0.007 L/mg) was higher than Mg-Al-Ni LDH (0.1819 ± 0.004 L/mg), at 20 °C. The final adsorption capacity was higher for Mg-Al LDH (91.85 ± 0.087%) in comparison with Mg-Al-Ni LDH (35.97 ± 0.093%), at 20 °C. It was found that the adsorption kinetics is best described by the pseudo-second-order model. The results indicated that LDHs can be considered as a potential material for adsorption of other metallic ions from wastewater.

## 1. Introduction

Pollution of water bodies by metals has received increasing public attention due to the importance of metals in industrial processes and their potential toxicity towards humans and in aquatic environments [1]. In the earth’s crust, manganese is frequently associated with iron. The main minerals are: Pyrolusite (MnO_2_), rhodocrosite (MnCO_3_), hausmanite (Mn_3_O_4_) and braunite (Mn_2_O_3_). Manganese oxides can be reduced with coal in furnaces at high temperatures or in electric furnaces. Pure manganese can be obtained by aluminothermy, by electrolysis of MnSO_4_ concentrated solution or by sodium dislocation of MnF_2_ or MnCl_2_ [2,3].

The sources of manganese pollution are: Siderurgy, an alloying element with other metals such as iron (Fe), nickel (Ni), copper (Cu) and aluminum (Al); manufactures of dry batteries, glass and products from the ceramics industry, chemical industry and paint industry. Wastewaters from industries, mainly the steel manufacturing industry (about 95%), are the main sources of manganese pollution, along with the processing and exploitation of ores [2,3].

There is an essential trace element in an organism’s manganese, its function being related to the metabolism of oxygen. The daily dose in humans is 5–10 mg. High doses of ingested manganese can cause local affections (burns of the digestive tract, vomiting, glottic edema and digestive bleeding) and general affections (liver, respiratory and cardiovascular disorders). Respiratory tract damage may occur during inhalation in the case of occupational exposures to iron and manganese processing, in the production of alloys, mining, welding and working with agrochemicals products. Neurological symptoms appear in chronic intoxication and develop slowly only after years of exposure. In this case can headache, asthenia, drowsiness, changes in gait “went of cock” or irreversible effects such as Parkinson’s syndrome, dysgraphia, dysarthria and psychic impairment appear. From US statistics, a population between 500,000 and 1,500,000 people has been diagnosed with Parkinson’s disease, and doctors are considering possible exposure to manganese [2]. In drinking water, the concentration of manganese should not exceed 0.1 mg/L. Legislation has established a maximum concentration of manganese in effluent of 1.0 mg/L [3].

Manganese has high solubility under both acid and neutral conditions, which is why it is considered a difficult element to remove from aqueous media [4]. The most used methods for the removal of manganese are precipitation processes with carbonates, hydroxides or oxidative precipitation [5]. Oxidation of Mn^2+^ to Mn^4+^ can be most easily done with air or O_2_ when manganese dioxide is formed, but the reaction has a slow speed. Other oxidation agents used in this process are potassium permanganate, hypochlorite or ozone [5], but these oxidations have the disadvantage of high costs and special methods of handling the reagents [6].

Various techniques for treating manganese wastewater have proved to be disadvantageous due to the following reasons: They generate secondary pollution and are inefficient in the case of low manganese content, and high costs are recorded [7]. Therefore, the researchers focused on discovering cheap, environmentally friendly, simple and efficient manganese removal methods. The layered double hydroxide (LDH) materials for removal of manganese can be synthesized in the laboratory by simple and economical methods (e.g., incipient impregnation [8], hydrothermal routes [9], dropwise addition of a chloride solution [10], hydrolysis [11], solar light irradiation [12], co-precipitation [13], ionic exchange, direct co-precipitation and structure reconstruction after calcination [14]). Wimonsong et al. [8] report the preparation of different Au/Fe-Zn-Mg-Al-O hydrotalcite (HT) supported Au catalysts by a co-precipitation method. The results reveal that the activity of Au highly depends on the support materials. Lin et al. [9] fabricated the three-dimensional CuCo_2_S_4_@NiMn LDH core-shell heterostructures on Ni foam via the hydrothermal routes directly for supercapacitor electrodes. They demonstrated that these core-shell hybrid arrays exhibit good rate capability with a long cycle lifetime. Kameda et al. [10] studied the equilibrium and kinetics in order to determine the effect of Fe^2+^-doped Mg-Al LDH on the removal of As(V) and Sb(V) from an aqueous solution. Sepehr et al. [11] synthesized magnesium (Mg)/aluminum (Al) layered double hydroxide (LDH) nanoparticles by hydrolyzing urea and used them to remove metronidazole (MN) from aqueous solution. The obtained results showed that the adsorption of MN onto LDH was strongly dependent on pH and weakly dependent on foreign ionic strength. Gilea et al. [12] prepared the heterostructures of silver nanoparticle (Ag)/zinc (Zn) based layered double hydroxides as efficient photocatalysts for degrading of 4-nitrophenol (4-NPh) from aqueous solutions, using solar light irradiation. Results showed that Ag/ZnAl LDHs heterostructures were active photocatalysts under solar light. Gu et al. [13] report a review that provides a systematic overview of LDH based materials and their application in radionuclide removal from aqueous solutions. Moreover, they present preparation and modification methods that offer an intuitive understanding of LDHs. Seftel et al. [14] prepared the layered double hydroxides (LDHs) containing Mg and Al or Zn and Fe by co-precipitation, using NaOH and urea as precipitation agents. The study was made regarding the possibilities of incorporation of an organic anion (phenyl-alanine amino acid anion) between the cationic layers, by using three different methods: Ionic exchange, direct coprecipitation and structure reconstruction after calcination at 400 °C. It is known that LDH is an active adsorbent, but studies on the effects of different parameters on the efficiency of eliminating Mn^2+^ have not been adequately explored. Figueiredo et al. [15] showed that the iron-based LDHs Zn_4_FeAl and Mg_4_FeAl, constituted by a high level of the endogenous iron ions, are potential candidates used in the therapeutic field. Benício et al. [16] synthesized and characterized layered double hydroxides (MgAl LDHs, MgFe LDHs and MgAlFe LDHs) containing phosphate anions. Results reveal that LDHs present structural and chemical characteristics that make them potential materials for use as storage matrices for slow P release. Zhao et al. [17] designed and fabricated NiMn layered double hydroxide (NiMn LDH) microcrystals grafted on a carbon nanotube (CNT) backbone by an in situ growth route. Electrochemical evaluation reveals that the NiMn LDH/CNT electrode delivers a high specific capacitance with good rate capability and excellent cycle-ability. Gutierrez Moreno et al. [18] showed that the amorphous silica (SiO_2_) shell on diatom frustules is a highly attractive biomaterial for removing heavy metal from aquatic ecosystems. The results indicate that the adsorption was strongly driven by van der Waals interactions at the highly covered surface, where the consideration of dispersion corrections reduces the modifier−adsorbate interatomic distances and increases the adsorption energy. Hernández-Ávila et al. [19] used different minerals from the country of Mexico to eliminate toxic cations from a synthetic solution containing As^3+^ (arsenic), Ag^+^ (silver), Ni^2+^ (nickel), Cr^6+^ (chromium) and Pb^2+^ (lead). The results indicate that the recovery of arsenic, silver, lead and nickel was up to 95% and for chromium was lower than 10%, for both minerals. Torres et al. [20] presented a study based on dissolution of copper from chalcopyrite using a wastewater with high chloride content and MnO_2_ present in manganese nodules as an oxidizing agent. They showed that a higher copper extraction can be obtained at a high temperature (80 °C) and at acid concentration (H_2_SO_4_) of 1 mol/L. Saeki et al. [21] investigated the adsorption of Fe^2+^ and Mn^2+^ on variable charge materials (silica, gibbsite and humic acids). The studies indicated that Fe^2+^ has a stronger adsorptive affinity to the oxides than does Mn^2+^.

In this paper, we present the synthesis and characterization of novel Mg-Al- and Mg-Ni-Al LDHs for the removal of Mn^2+^ from synthetic wastewater. The adsorption equilibrium on these LDHs was investigated at different experimental conditions such as: Shaking time, initial Mn^2+^ concentration and temperatures (10 and 20 °C). The structural and morphological properties of LDHs were investigated by X-ray diffraction (XRD), Scanning Electron Microscopy (SEM) and Energy Dispersive Spectrometry (EDX) analysis. Adsorption isotherms of Mn^2+^ were fitted by Langmuir and Freundlich models. The environmentally friendly procedure was chosen for the synthesis of LDHs because it is a procedure with low cost, it uses eco-friendly materials and it could potentially be applied on an industrial scale.

## 2. Materials and Methods

Mg(NO_3_)_2_·6H_2_O, Al(NO_3_)_3_·9H_2_O, Ni(NO_3_)_2_·6H_2_O, Na_2_CO_3_, NaOH and MnSO_4_ were purchased from Sigma Aldrich (Merck KGaA, Darmstadt, Germany) and used without further purification. H_2_SO_4_ was supplied by Merck (Merck KGaA, Darmstadt, Germany). All reagents used were analytical grade. Distillated water was used for the preparation of materials and synthetical wastewater.

### 2.1. Synthesis of the Mg-Al- and Mg-Ni-Al LDH Materials

The Mg-Al and Mg-Ni-Al LDH materials were synthesized by the chemical co-precipitation method at constant pH = 8.45 (pH-meter JK-PH009, JKI, Shanghai, China). In order to synthesize the Mg-Al LDH, a typical procedure was followed: A stoichiometric amount of reagents Mg(NO_3_)_2_·6H_2_O and Al(NO_3_)_3_·9H_2_O was dissolved in distilled water (total concentration 1 M; the cationic ratio Mg^2+^:Al^3+^ was 3:1). The obtained solution was added slowly over a Na_2_CO_3_ solution (2 × 10^−3^ M) under vigorous stirring (200 rpm). During the synthesis, the pH value was kept constant at 8.45 by adding of NaOH solution (0.1 M). After that, the obtained mixture was stirred continuously for 30 min at room temperature (22 ± 1 °C). The formed precipitate was left to mature for 12 h, then filtered, washed with distilled water several times and dried in air at room temperature. Precipitate was calcined at 750 °C for 8 h using an oven. After that, the obtained powder was left to dry at room temperature for 8 h, then used for adsorption of manganese ions. A similar procedure was employed for the synthesis of Mg-Ni-Al LDH, using Mg(NO_3_)_2_·6H_2_O, Al(NO_3_)_3_·9H_2_O and Ni(NO_3_)_2_·6H_2_O (total concentration 1 M; the cationic ratio Mg^2+^:Ni^2+^:Al^3+^ was 2:1:1). Calcination temperature was 750 °C.

**Figure 1:**
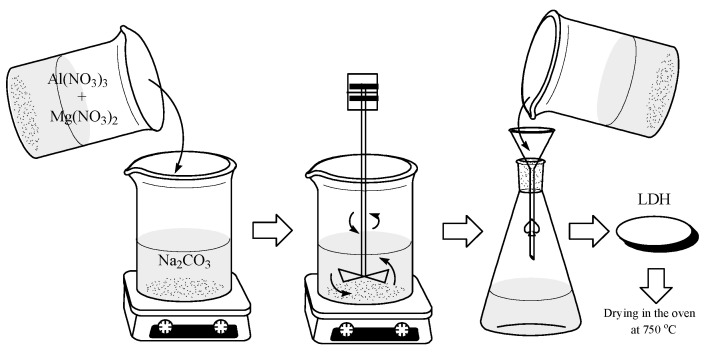
Schematic pathway to obtain layered double hydroxides (LDHs).

A schematic pathway to obtain LDHs is shown in Figure 1.

### 2.2. Characterization of the Synthesized Materials

Structural properties of the Mg-Al LDH and Mg-Al-Ni LDH, before and after adsorption of Mn^2+^, were observed by Energy Dispersive Spectrometry (EDX) analysis. The phase composition analysis for materials was performed with the X-ray diffraction method using a Panalytical X’PERT Pro MPD X-ray diffractometer with 2θ Bragg–Brentano geometry (Shimadzu, Duisburg, Germany). An X-ray beam characteristic to Cu Kα radiation was used (λ = 1.5418 Å). The experimental data were digitally collected by the “step by step” scanning method in the 2θ range of 5 ÷ 80 degrees.

Surface morphology of LDHs, including microstructure and elemental micro-composition, was performed by Scanning Electron Microscopy (SEM) analysis coupled with Energy Dispersive X-ray Spectrometry (EDXS). The analysis was performed using a Quanta Inspect F50 SEM device (Hitachi, Berkshire, United Kingdom), equipped with a field emission gun (FEG) with 1.2 nm resolution and coupled with an EDAX spectrometer with 133 eV resolution at Mn Kα.

Textural analysis was made using Micromeritics ASAP 2020 (Micromeritics, Unterschleissheim, Germany) by adsorption of nitrogen at the liquefaction temperature. The samples were first degassed for 2 h at 150 °C and 0.1 Pa and then subjected to analysis in order to determinate the specific surface area, the pore volume and the mean pore size.

The obtained LDHs (see Table 1) were examined before (blank) and after adsorption of Mn^2+^.

### 2.3. Adsorption Study of Mn^2+^ Used LDHs

Mixed oxides Mg-Al and Mg-Al-Ni LDHs were used in order to establish the equilibrium conditions for removal of Mn^2+^ from synthetic wastewaters. Stock solutions that contained Mn^2+^ were prepared by dissolving MnSO_4_ in distillated water at room temperature (22 ± 2 °C). The working solutions were prepared by diluting the stock solution with distillated water and H_2_SO_4_, used to adjust the pH.

For the dynamic studies, the experimental procedure was: 0.3 g of mixed oxides Mg-Al and Mg-Al-Ni LDHs, separately, were placed in Berzelius glasses, where 50 cm^3^ of MnSO_4_ solution, with various concentrations, was added: 2 mg/L, 5 mg/L, 10 mg/L, 20 mg/L, 40 mg/L, 60 mg/L, 80 mg/L and 100 mg/L. Glasses were coated in order to prevent solution evaporation. Mixed oxide LDHs were in contact with the solution for 24 h then filtered using blue ribbon paper and the solution was placed in a volumetric flask of 50 mL. From the filtrate, a 10 cm^3^ solution was spectrophotometrically analyzed at λ = 525 nm (Shimadzu 1900 UV-VIS, Tokyo, Japan). LDHs were used as powders.

For the kinetic studies, the experimental procedure was: 0.3 g of mixed oxide LDHs was placed in Berzelius glasses, where 50 cm^3^ of MnSO_4_ solution with concentration of 100 mg/L was added. Glasses were coated in order to prevent solution evaporation and were stirred (200 rpm) at different times (5, 10, 20, 30, 60 and 90 min) and temperatures (10 and 20 °C).

**Figure 2:**
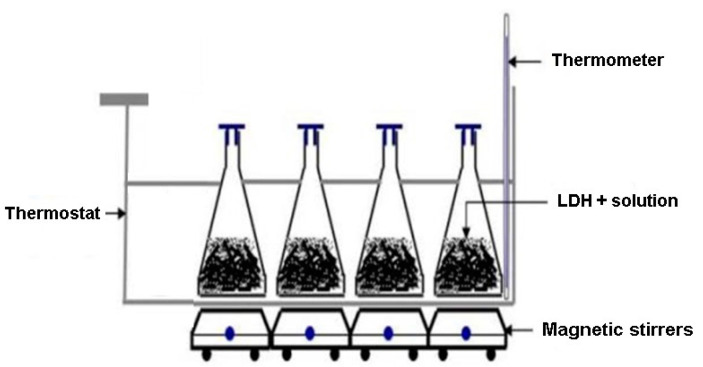
Adsorption study of Mn^2+^ using LDH.

Magnetic stirring was realized at 300 rpm, using a Heidolph Unimax shaker (Heidoph, Schwabach, Germania). At the end of stirring, the samples were filtered using blue ribbon paper and the solution was placed in a 50 mL volumetric flask (Figure 2). From the filtrate, 10 cm^3^ of solution was spectrophotometrically analyzed at λ = 525 nm. The procedure for determination of Mn^2+^ concentration from solutions was performed three times and data are presented as means ± standard deviation. All experiments were made at pH = 7 (pH-meter JK-PH009, JKI, Shanghai, China, 0–14 pH). The Mn^2+^ removal percentage (R%) was calculated using Equation (1) [22,23]:

(1) $$R\%=\frac{C_{i}-C_{e}}{C_{i}}\times 100$$

where: C_i_—initial concentration of Mn^2+^ (mg/L); C_e_—Mn^2+^ concentration in the aqueous phase, at equilibrium (mg/L).

#### 2.3.1. Adsorption Isotherms

Langmuir [22,24] and Freundlich [25,26] models are most commonly models used especially for adsorption processes in water or wastewater treatment.

The Langmuir model is expressed in Equation (2) [22,24]:

(2) $$a=\frac{K_{L}\cdot C_{e}\cdot a_{\max}}{1+K_{L}\cdot C_{e}}$$

where: a—adsorption capacity of LDH at equilibrium (mg/kg); K_L_—Langmuir equilibrium constant of the adsorption process (L/mg); C_e_—Mn^2+^ concentration in the aqueous phase, at equilibrium (mg/L); a_max_—maximum adsorption capacity of LDH (mg/kg).

The Freundlich adsorption isotherm was used in case of heterogeneous adsorbent. In literature [25,26], the isotherm interprets that the ratio of the ion amounts adsorbed on a known mass of adsorbent to the ion concentration in the solution differs at different concentrations [22,27]. The Freundlich model starts from the hypothesis of reaching the chemical equilibrium when there is a dynamic exchange between the molecules of the adsorbed phase and those remaining in solution [25,27].

Freundlich adsorption isotherm is expressed in Equation (3) [22,25]:

(3) $$\ln q_{e}=\ln K_{F}+\frac{1}{n}\ln C_{e}$$

where: C_e_—Mn^2+^ concentration in the aqueous phase, at equilibrium (mg/L); q_e_—adsorbent equilibrium concentration (mg/g); 1/n = m—the constant indicating the Freundlich isotherm curvature; K_F_—Freundlich equilibrium constant ((mg/g)/(mg/L)^n^).

#### 2.3.2. Kinetics Studies

Pseudo-first order and pseudo-second order were used to evaluate the kinetic adsorption data. Linear forms of these kinetic models are presented in Equations (4)–(6) [23,27].

(4) $$\ln\left(q_{e}-q_{t}\right)=\ln q_{e}-k_{1}t,$$

(5) $$\frac{t}{q_{t}}=\frac{1}{k_{2}q_{e}{}^{2}}+\frac{t}{q_{e}}$$

(6) $$q_{t}=K_{id}t^{1/2}+C$$

where: q_e_ and q_t_—adsorption capacity at equilibrium and at time *t*, respectively, (g Mn^2+^/g of sorbent); k_1_—pseudo-first-order rate constant (min^−1^); k_2_—pseudo-second-order rate constant (g mg^−1^ min^−1^); K_id_—diffusion rate coefficient (g mg^−1^ min^−1^); C—intercept, related to the thickness of the boundary layer.

## 3. Results and Discussion

### 3.1. Characterization of LDHs

**Figure 3:**
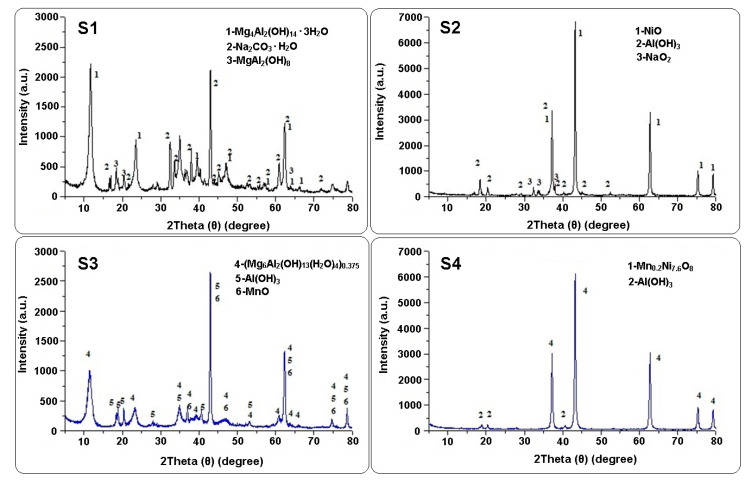
XRD patterns for LDHs before and after Mn^2+^ adsorption.

XRD analysis was performed in order to observe the interaction of Mn^2+^ with LDHs. Figure 3 showed the XRD spectra of LDHs before and after Mn^2+^ adsorption.

The XRD patterns of LDHs, before and after Mn^2+^ adsorption (Figure 3), indicated that the intensity of peaks decreases and the basal distance increases after reaction, indicating the incorporation of manganese species into the interlayer of the LDHs. The XRD patterns indicated that the prepared LDHs are anionic layered materials. This means that they consist of positively charged layers (brucite-like layers) and the charge of the layers is compensated by interlayer anions (typically carbonates). The increase in the interlayer distance could be due to the deposition of larger anions (such as hydroxide and carbonate), changing of anion orientation in the interlayer space or accommodation of more water molecules into the interlayer space. The existence of partly intercalated LDH particles could be due to the restricted intercalation reaction and the presence of carbonate ions (CO_3_^2−^) in the structure of LDHs. Therefore, the prepared Mg-Al LDH is a double layered hydroxide containing carbonate.

As indicated in Figure 3, the XRD pattern for prepared LDHs, before adsorption, presented sharp and symmetrical peaks and some high-angle asymmetrical peaks, demonstrating the formation of a highly crystalline Mg-Al LDH structure. It was reported in the literature that most CO_3_^2−^containing LDH have a crystalline structure because the prismatic arrangement of hydroxyl groups allows those in both lower and upper layers to form hydrogen bonds with the oxygen atoms of CO_3_^2−^ [26]. The XRD results show that the diffraction intensity of peaks observed at 2θ = 42.12° increases after Mn^2+^ adsorption. This fact could indicate that the interlayer distance of Mg-Al LDH decreased. 

The 2θ and d-spacing values of LDHs before and after adsorption of Mn^2+^ are listed in Table 2. The lattice parameters (a, b, c; α, β, γ) calculated from the XRD data describe the size and shape of the unit cell (smallest repeating unit of the crystal lattice; a represents the average cation–cation distance within the brucite-like layers; b and c are related to the thickness of the brucite layer and the interlayer distance) [28].

The phases encountered for the sample S1 were: Na_2_CO_3_.H_2_O—50% crystallized in the orthorhombic system (space group: Pnma); Mg_4_Al_2_(OH)_14_.3H_2_O—47% crystallized in the rhombohedral system; and MgAl_2_(OH)_8_—43% crystallized in the monoclinic system. The phases encountered for the sample S2 were: NiO—62% with cubic crystallization (space group: Fm-3m) and the sides of 4.18 Å; Al(OH)_3_—40% crystallized in monoclinic system (space group: P21/c); and NaO_2_—33% crystallized in orthorhombic system (space group: Pnnm). The Mg compounds were not identified in this analysis. The phases encountered for the sample S3 were: MnO—58% crystallized in the cubic system (space group: Pnnm); Al(OH)_3_—56% crystallized in the hexagonal system (space group: Pnnm)—which is in fact Al(OH)_3_; and (Mg_6_Al_2_(OH)·18(H_2_O)_4_)_0.375_ (hydroxide double Mg and Al hydrated)—49% crystallized in rhombohedral system. A change can be observed in the crystal lattice exhibited by increasing the value of the interlayer distance of the crystalline planes of the peak from 2θ = 11.66°, from the value of d = 7.581 Å for the sample S1, in the case of compound Mg_4_Al_2_(OH)_14._3H_2_O, at the value d = 7.74 Å in position 2θ = 11.42° of the sample S3, in the case of compound ((Mg_6_Al_2_)(OH)_18_(H_2_O)_4_)_0.375_. Both compounds have a rhombohedral system with space group R-3m. The lattice parameter (c) of the crystal system for the compound Mg_6_Al_2_(OH)·18(H_2_O)_4_)_0.375_ indicated that the interlayer distances of the compound were greater (22.932 Å and 23.028 Å). In the case of sample S4 two phases were indicated: β-Al(OH)_3_—25% crystallized in monoclinic system (space group: C2/m); and a mixed oxide of Mn and Ni—75% crystallized in cubic system.

The compound Mn_0.2_Ni_7.6_O_8_ identified in the sample S4 is crystallized in a cubic system, with space group Fm-3m, as well as the compound NiO from the sample S2. The network of the compound NiO in the sample S2 had the interlayer distance d = 8.356 Å for 2θ = 43.25°, and the compound Mn_0.2_Ni_7.6_O_8_ in the sample S4 had the interlayer distance d = 8.392 Å for the same 2θ = 43.25°. The NiO network changed, possibly due to the presence of Mn^2+^ ions in the network of the new compound formed, Mn_0.2_Ni_7.6_O_8_. The interlayer distance of the formed compound was also higher than the interplanar distance calculated for 2θ = 43.31°, which was d = 8.349 Å. It is possible to form the compound Mn_0.2_Ni_7.6_O_8_ by replacing some Ni^2+^ with Mn^2+^.

Sepehr et al. [11] characterized magnesium/aluminum layered double hydroxide (LDH) nanoparticles after adsorption of metronidazole from aqueous solution. The XRD results show that the peak observed at 2θ = 10.49° was shifted to a stronger angle after metronidazole (MN) adsorption. This indicated that the interlayer distance of Mg-Al LDH decreased. Teixeira et al. [25] studied the removal of manganese and fluoride from industrial wastewaters (IW) using layered double hydroxides (LDHs). They synthesized Mg-Al LDH material by a chemical co-precipitation method and ternary Mg-Mn-Al LDHs (LDH-IW) from IW containing a high concentration of manganese. The XRD patterns for both types of material exhibited the typical planes of hydrotalcite, with high crystallinity and purity. The results show that the basal spacings for LDH were 7.56 Å and LDH-IW was 7.59 Å, respectively, and the crystallite sizes of the samples were 15 and 12 nm for LDH and LDH-IW, respectively. Yang et al. [26] synthesized Mg-Al and Zn-Al layered double hydroxides (LDHs) for removal of phosphate from the natural water or wastewater treatment. The XRD patterns of Mg-Al and Zn-Al LDHs shows strong peaks and a series of peaks that appeared as sharp and intense symmetric lines. These indicate the characteristic basal reflections of hydrotalcite-like LDH materials and a large distance between interlayers. Shi et al. [29] fabricated a humic acid/MgAl layered double hydroxide composite (HA/MgAl LDH) for the removal of cadmium in aqueous solution. The results indicated that the pure MgAl LDH has a diffraction peak at 60.72° that can be attributed to the plane of Mg_4_Al_2_(OH)_12_CO_3_·3H_2_O. The diffraction peak at 46.99° was slightly lower for HA/Mg-Al LDH in comparison with pure Mg-Al LDH. This was attributed to the addition of heat-treated humic acid.

**Figure 4:**
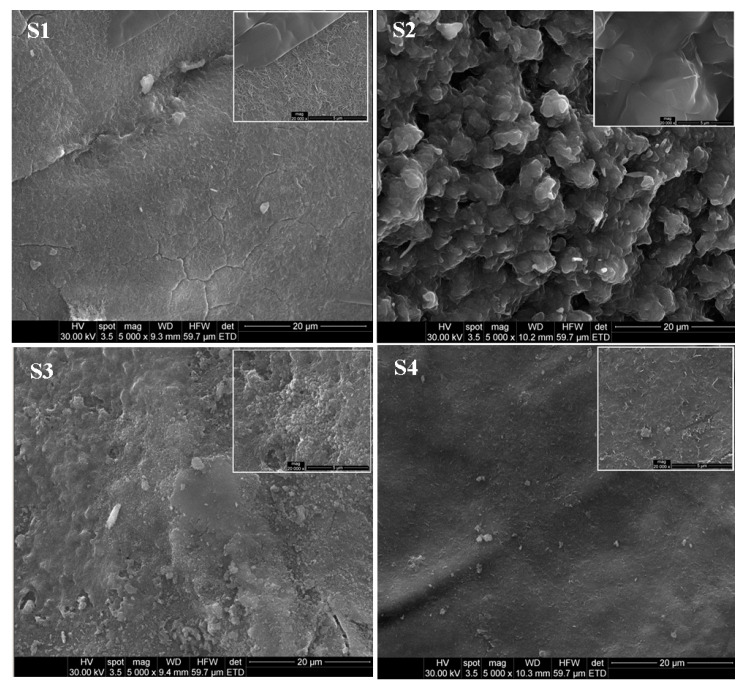
SEM images for LDHs, before (samples S1 and S2) and after (samples S3 and S4) Mn^2+^ adsorption.

Surface morphology of LDHs before (samples S1 and S2) and after Mn^2+^ adsorption (samples S3 and S4) was observed by SEM at 5000 × magnifications (Figure 4).

It can be observed from Figure 4 that the Mg-Al LDH and Mg-Al-Ni LDH exhibit agglomerates with compact and non-porous structure. The LDH particles are uniformly shaped sheets, most of them being hexagonal. After adsorption, LDH materials present several heterogeneous small pores, which are known to provide larger surface area for contaminant adsorption. The introduction of the Ni ion into the Mg-Al LDH structure could create more exterior hydroxyl ions (OH^−^). This could form chemical precipitates which lead to a reduction in the adsorption capacity. In the case of sample S4, the present elements were uniformly distributed. The particles observed in the SEM image are not precipitated, but they are small fractions of samples that remain on the analyzed surface. Sepehr et al. [11] showed that the surface area of the magnesium/aluminum layered double hydroxide (LDH) nanoparticles was 109 m^2^/g. Moreover, the surface morphology images of the LDH nanoparticles revealed that the adsorbent surface consisted of hexagonal nanosheets with a diameter between 200 and 1000 nm. Teixeira et al. [25] related that the morphology images for synthesized Mg-Al LDH and ternary Mg-Mn-Al LDHs (LDH-IW), exhibited the agglomerates with different sizes. The LDH-IW exhibited high crystallinity and structural properties like those of LDH, although the particle size was smaller. The results were in accordance with the XRD analysis, where the calculated particle sizes were 15 nm (LDH) and 12 nm (LDH-IW), respectively.

**Figure 5:**
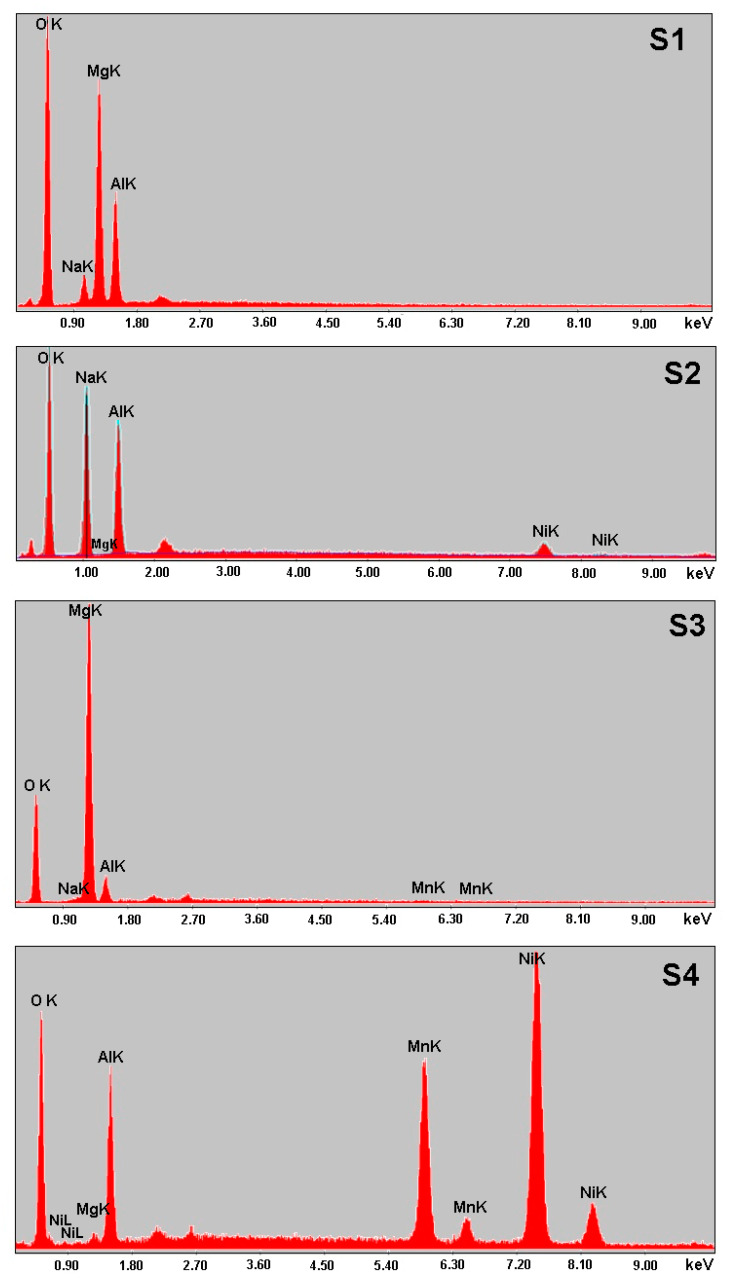
EDX surface analysis of the LDHs, before and after the Mn^2+^ adsorption.

EDX analysis was performed in order to reveal the interaction of manganese with LDHs. Figure 5 shows the EDX spectra of LDHs, before and after Mn^2+^ adsorption.

EDX illustrated that Mg-Al LDH (sample S1) was composed of Mg, Na, O and Al. The calculated quantitative percentages Na 4.52%, Mg 26.50%, Al 16.67% and O 52.31% demonstrate the unevenness of the material. The EDX for Mg-Al LDH (sample S2) showed that the sample has uniformly spread elements. The Mg is not identified, but the following elements are detected: Na 30.81%, Al 22.25%, Ni 3.97% and O 42.97%. The higher percentage of Na and O indicated that NaO_2_ is present in a large quantity. The lack of Mg can be explained by the inclusion of Mg in certain compounds such as aluminum hydroxide and sodium oxide. The EDX for Mg-Al LDH Mn^2+^ (sample S3) indicate the presence of Mg, Al, Na, Mn and O. The calculated quantitative percentages of elements detected were: O 39.52%, Na 0.65%, Mg 50.63%, Al 8.56% and Mn 0.63%. In the case of Mg-Al-Ni LDH Mn^2+^ (sample S4), the deposit of a quantity of Mn (14.75%) and a small quantity of Mg (1.23%) was observed. The absence of a peak for Mg can be attributed to complexation of Mg with oxygen atoms. Na was not detected in the sample, but O 24.22% and Al 17.15% were detected. The absence of a peak for Na can be attributed to complexation of Na with oxygen atoms. The Ni peak observed at 7.39 keV can indicate that the Mn was adsorbed on Mg-Al-Ni LDH (sample S4) for 42.64%. This could indicate that the Ni favors the retention of Mn and the compound identified by XRD analysis being a mixed oxide of the Mn_0.2_Ni_7.6_O_8_ type. The peaks of Al for Mg-Al LDH slightly changed from 1.56 keV before adsorption (sample S1) to 1.63 keV after adsorption (sample 3) possibly due to the formation of a Mn-O bond. The peaks of O, before and after adsorption of Mn^2+^, could correspond to carbonyl groups, Mg/Al-O bonds, Mg/Al/Ni-O bonds, hydroxy groups and carboxy groups.

Shi et al. [29] studied the surface chemical characteristics of Cd-loaded and unloaded HA/MgAl LDH by XPS to further confirm the adsorption mechanisms of Cd by the HA/MgAl LDH composites. The XPS spectra illustrated that HA/MgAl LDH was composed of Mg, Al, C, O and N. The Cd peaks observed at 405 eV and 411.74 eV indicated that the Cd was adsorbed on HA/MgAl LDH for 59.97% and 40.03%, respectively, indicating that the binding of Cd is dominated by surface complexation.

### 3.2. Adsorption Isotherms and Kinetics Evaluation

#### 3.2.1. Adsorption Isotherm Evaluation

Adsorption capacity of LDHs for Mn^2+^, at different aqueous equilibrium concentrations and temperatures (10 and 20 °C), was illustrated by the adsorption isotherms. The distribution of manganese between the liquid and the solid phase in equilibrium is expressed by the Freundlich and Langmuir models. The fitted constants for the two equilibrium models along with regression coefficients are summarized in Table 3.

**Figure 6:**
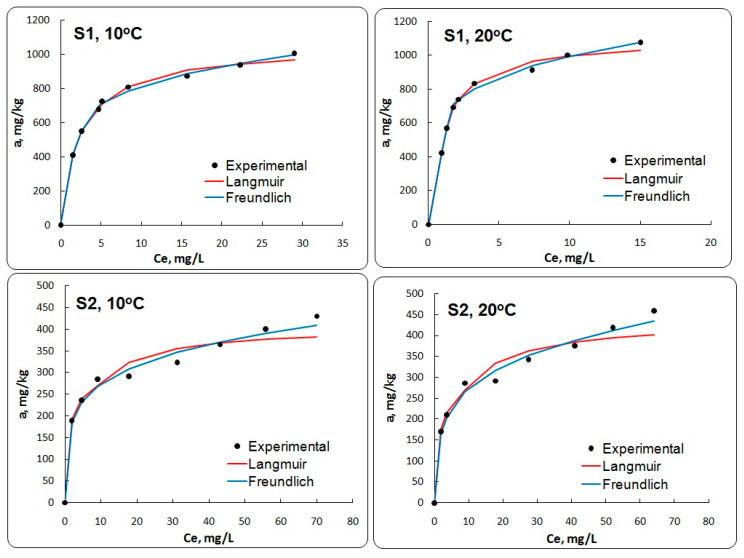
The adsorption curves of Mn^2+^ using Mg-Al LDH (sample S1) and Mg-Ni-Al LDH (sample S2), at 10 and 20 °C.

The adsorption isotherm of Mn^2+^ on Mg-Al- and Mg-Al-Ni LDHs, measured with the initial Mn^2+^ concentration ranging from 2 to 100 mg/L, is shown in Figure 6. It can be observed that the adsorption capacity of Mn^2+^ increased with the increasing initial Mn^2+^ concentration. Figure 6 shows the validity of Freundlich adsorption isotherm in the adsorption process of Mn^2+^ on Mg-Al LDH, at different conditions. In the case of Mg-Al-Ni LDH (sample S2), the increase of equilibrium adsorption capacities of the Mn^2+^ adsorption process by increasing the temperature indicates that the process occurred according to chemical adsorption (chemisorption) [22,30].

The results indicate that Mg-Al LDH (sample S1) could be a promising adsorbent. The adsorption onto Mg-Al LDH could be described by the Freundlich model, which was attributed to the greater heterogeneity of the Mg-Al LDH surface. The difference in the adsorption of Mn^2+^ by increasing the temperature could be attributed to the increase in the adsorbent porosity. At 60 min of shaking time, 15% and 90% of Mn^2+^ were removed for the initial concentration.

Final removal percentage of Mn^2+^ was higher for Mg-Al LDH (sample S1) (91.85 ± 0.087%) in comparison with the Mg-Al-Ni LDH (sample S2) (35.97 ± 0.093%), at 20 °C. The presence of nickel reduces the number of free active centers on the surface of Mg-Al-Ni LDH (sample S2), thus preventing the adsorption of manganese ions. This highlights the fact that, in the case of sample S2, the dominant process is due to the chemical precipitation, which leads to a reduction in the adsorption capacity. For this reason, the experimental data do not fit the curves of the model. This fact is also highlighted by the value of R% which, for the Mg-Al LDH (sample S1), is higher in comparison with Mg-Al-Ni LDH (sample S2), which can be used in the removal of other metallic ions from wastewaters.

Yang et al. [26], indicated that the removal ratio of phosphate by Mg-Al and Zn-Al layered double hydroxides (LDHs) gradually increased in the first 20 min and reached equilibrium at about 40 min. The final adsorption efficiencies for both types of LDH were higher than 95%. Shi et al. [28] showed that Cd was absorbed on the surface of humic acid/MgAl layered double hydroxide (HA/MgAl LDH) by surface complexation (59.97%) and chelating interaction (40.03%).

The adsorption isotherm parameters for Mg-Al LDH and Mg-Al-Ni LDH are presented in Table 3.

The Langmuir constant (K_L_) related to the affinity between the adsorbent and adsorbate, and the Freundlich constant (K_F_) related to the adsorption capacity of adsorbent, indicative of affinity between the species, and m is the constant related to sorption intensity, indicative of the metal concentration effect. Based on the obtained results (Table 3), the K_L_ value of Mg-Al LDH (0.9529 ± 0.007 L/mg) was higher than Mg-Al-Ni LDH (0.1819 ± 0.004 L/mg), at 20 °C. The high-energy sites with high constant (K_L_) had an important higher affinity than low-energy sites with a low constant (K_L_) [28]. Increasing the temperature of the adsorption process increases the adsorption capacity of the Mg-Al LDH [22,29]. The maximum adsorption capacities (a_max_) of prepared LDHs are summarized in Table 3. The a_max_ value of Mg-Al LDH (1100.607 ± 0.656 mg/kg) was higher than Mg-Al-Ni LDH (436.972 ± 0.260 mg/kg), at 20 °C. The results demonstrate that the prepared Mg-Al LDH exhibited an excellent adsorbent capacity for Mn^2+^ (1074.923 ± 0.521 mg/kg, initial Mn^2+^ concentration of 100 mg/L) compared to other adsorbents such as kaolinite (0.446 mg/g) [31], *Riccia xuitans* (0.254 mg/g) [32], *Bombax malabaricum* (0.2837 mg/g), *Ipomea batatas* (0.3627 mg/g), *Pithecelobium dulse* (0.4156 mg/g) and *Peltaforum ferraginium* (0.4433 mg/g) [33].

As shown in Table 3, the isotherm data were better fitted into the Freundlich model than the Langmuir model. The values show that the Freundlich model offers the best approximation of the retention isotherms of Mn^2+^ (R^2^ = 0.9096 ÷ 0.9808) for both types of sample (because this model assumes that the adsorbent surfaces are inhomogeneous in terms of energy) in comparison with the Langmuir model (R^2^ = 0.7282 ÷ 0.9759) (which starts from the hypothesis that the adsorbent surfaces are homogeneous). The higher values of R^2^ indicate that the Freundlich model was more suitable for describing the adsorption equilibrium of manganese onto the LDH adsorbent. The manganese adsorption data obtained were better represented by the Freundlich model than by the Langmuir model, as confirmed by the R^2^ values, at a temperature of 20 °C. This fact can indicate that the manganese monolayer adsorption on the surface of the adsorbent was heterogeneous. The results indicate that there may be a higher affinity between Mn^2+^ and the available sites and well distributed active sorption sites of Mg-Al LDH beads, which leads to a faster adsorption.

Yang et al. [26] studied the adsorptive removal of phosphate by Mg-Al and Zn-Al layered double hydroxides. The R^2^ values obtained from the Freundlich and Langmuir isotherms for Mg-Al LDH were 0.998 and 0.990 and for Zn-Al LDH were 0.988 and 0.999, respectively. These results indicate a very good mathematical fit by both models. The equilibrium constant K_L_ value of Zn-Al LDH (0.634 L/mg) was higher than Mg-Al LDH (0.248 L/mg). Based on the values of a_max_ calculated by the Langmuir equation, the phosphate adsorption capacity for Zn-Al LDH (68.4 mg/g) was higher than that of Mg-Al LDH (31.3 mg/g). Modrogan et al. [27] synthetized a magnetic hydrogel adsorbent based on gellan gum and Fe_3_O_4_ for Mn^2+^ adsorption from groundwater. The results indicated that the maximum adsorption capacity was 21.739 mg/g. Shi et al. [29] synthetized the humic acid/MgAl layered double hydroxide composite (HA/MgAl LDH) and used it as an adsorbent for the removal of Cd in aqueous solution. The results indicate that the adsorption dynamics of HA/MgAl LDH followed a pseudo-second-order model, suggesting that the process of Cd removal was chemical adsorption. Adsorption isotherms followed the Langmuir model of monolayer adsorption, and the maximum theoretical adsorption capacity calculated for Cd was 155.28 mg/g. Li et al. [34] synthesized Mn-doped MgAl layered double hydroxides (LDHs) for the removal of arsenate from the aqueous solution. The study indicated that the adsorption capacity of As(V) on Mn LDHs via the Langmuir isotherm model was 166.94 mg/g.

Bakr et al. [22] studied the removal of Mn(II) from aqueous solutions by a Mg-Zn-Al LDH/montmorillonite nanocomposite. The results indicated that the maximum adsorption efficiency were 24.5, 26.4 and 28.9 mg/g at adsorbent mass of 0.25 g/L, initial Mn(II) concentration of 80 mg/L, pH of 6.0, stirring rate of 160 rpm, contact time of 75 min and temperatures 298, 308 and 318 K. The obtained results indicated that increasing the temperature of the adsorption process increases the adsorption capacity due to the acceleration of some original slow adsorption steps. This could be due to the new adsorption active sites that are created on the adsorbent surface or to the increases of the solubility of the Mn(II) ions in the medium [22,23].

#### 3.2.2. Kinetics Evaluation

**Figure 7:**
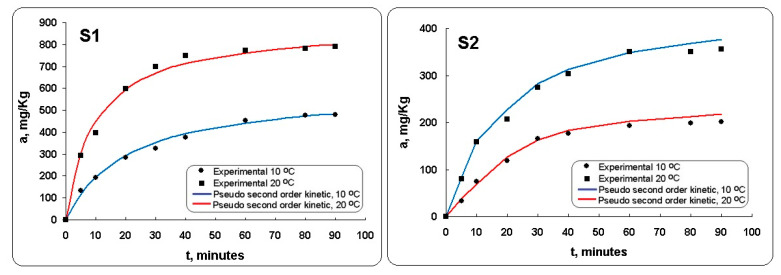
Integral kinetic curves for Mn^2+^ retention on Mg-Al LDH (sample S1) and Mg-Al-Ni LDH (sample 2), at 10 and 20 °C.

To understand the adsorption behavior of Mn^2+^ on Mg-Al LDH and on Mg-Al-Ni LDH, the Mn^2+^ adsorption kinetics process was presented in Figure 7 and evaluated by the pseudo-first-order model and pseudo-second-order model [22,27].

The adsorption as a function of contact time was conducted at different temperature. It can be observed (Figure 7) that in all experiments the adsorption equilibrium reached occurs in approximately 60 min. The curves showed that the adsorption capacity of Mn^2+^ was gradually increased with the contact time. In the initial stage, it can be observed that the adsorption capacity rapidly increases. This fact could be associated with the abundant reactive sites. As contact time increases further, after 60 min, the accessibility of the Mn^2+^ to unoccupied active sites on the adsorbent surface gradually decreases. This can be explained by the fact that these sites ultimately become saturated when the process reaches its equilibrium [35]. In case of the Mg-Al-Ni LDH (sample S2) the values of adsorption capacities with increasing of contact time were lower in comparison with those of the Mg-Al LDH (sample S2). This fact is specific to chemisorption and physical adsorption, where the adsorption is performed in a monomolecular layer without any interaction between the adsorbed ions. This also could be due to the adsorbed molecules that are linked to the surface by valence bonds. These molecules will usually occupy certain adsorption sites on the surface of Mg-Al-Ni LDH (sample S2) and only one layer of chemisorbed molecules will be formed (monolayer adsorption).

The kinetic parameters and the correlation coefficients are presented in Table 4.

As can be seen from Table 4, the correlation coefficients (R^2^) of the pseudo-first-order kinetic model were lower than the correlation coefficients of the pseudo-second-order kinetic model. The obtained data validate the pseudo-second-order kinetic model. This revealed that the rate determining step of Mn^2+^ adsorption is the chemical interaction between the metal ions and the LDHs. The process involves sharing of electrons between the metal ions and the negatively charged adsorptive centers (hydroxyl and carboxylic groups) of the LDHs. The results obtained from adsorption kinetics experiments, being in accordance with the pseudo-second-order equation, suggested a chemisorption process [22,30]. The adsorption capacities at equilibrium (q_e_) increase when the temperature increases for the Mg-Al LDH. This indicates that the process occurred according to chemical adsorption (chemisorption). It may be concluded that Mn^2+^ removal is done through a chemical reaction based on the pseudo-second-order model. This model suggests that the process involves chemical reaction mechanism [30].

Bakr et al. [22] applied the pseudo-first-order and pseudo-second-order models to evaluate the adsorption kinetics of Mn(II) ions onto the Mg-Zn-Al LDH/montmorillonite nanocomposite. The results obtained indicated that the adsorption of Mn(II) was governed by the pseudo-second-order kinetic model. This is confirmed by the correlation coefficients ranging between 0.996 and 0.999.

Yang et al. [26] indicated that the adsorption of phosphate onto Mg-Al and Zn-Al layered double hydroxides was governed by the pseudo-second-order kinetic model. This kinetic model indicates that the chemical adsorption or chemical bonding between adsorbent active sites and phosphate could dominate the adsorption process. The values of q_e_ for Mg-Al and Zn-Al LDHs were 9.78 mg/g and 24.8 mg/g, respectively. These values indicated that the phosphate adsorption capacity follows the order: Zn-Al LDH > Mg-Al LDH. Shi et al. [29] evaluated the adsorption process of cadmium using heat-treated humic acid/MgAl layered double hydroxide composite. Their experiments were fitted using different kinetic models, including the pseudo-first-order, pseudo-second-order and intraparticle diffusion models. The results show that, in all the fitting models, the correlation coefficient of the pseudo-second-order dynamics was up to 0.998.

### 3.3. Adsorption Mechanisms

**Figure 8:**
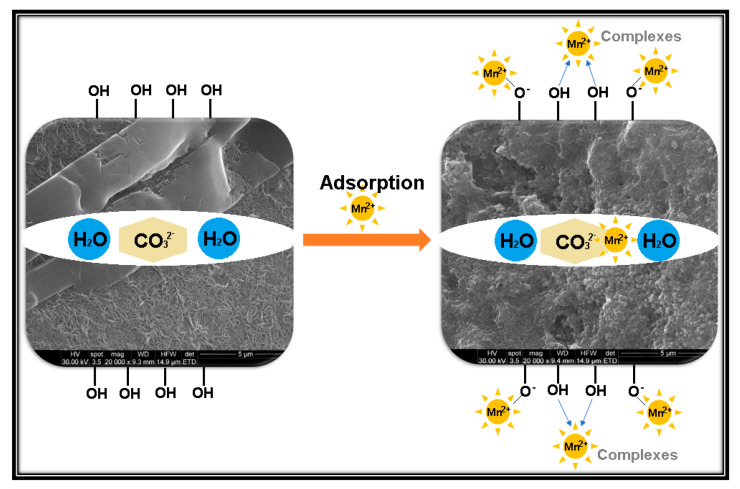
Schematic representation of the possible adsorption mechanism of Mn^2+^ onto Mg-Al LDH.

The possible adsorption mechanisms of Mn^2+^ onto Mg-Al LDH are schematically laid out in Figure 8.

The several processes take place simultaneously in the removal of Mn^2+^, such as adsorption, chemisorption, precipitation, co-precipitation, surface complexation and chelation, but the adsorption process is predominant, especially for the Mg-Al LDH.

The mechanism of adsorption Mn^2+^ on Mg-Al LDH, demonstrated by XRD and EDX analysis, is a complex process because every ion (Mn^2+^, CO_3_^2−^) presents different behavior when it is adsorbed on Mg-Al LDH [36]. The formation of the Mg-Al-CO_3_ LDH could improve the adsorption competence of Mn^2+^. The presence of hydroxyl groups attached to Mg-Al metal atoms on the outside of LDHs and a hydroxide ion in the region of the LDHs could determine a higher adsorption capability of LDHs for Mn^2+^. The favorite adsorption mechanisms among Mn^2+^ and Mg-Al-CO_3_- might contain the formation of MnCO_3_ and the creation of outer-sphere surface complexes with oppositely charged exterior hydroxyl groups (OH^–^) or with deprotroned hydroxyl groups (–O^−^), which could form complexes with metal cations [37]. Surface adsorption could involve the attachment of contaminants to the surface of LDHs, thus allowing the formation of a molecule or a film.

## 4. Conclusions

The present study intended to synthetize, characterize and apply novel Mg-Al- and Mg-Al-Ni LDHs for removing Mn^2+^ from synthetic wastewaters like groundwater. Stirring time, pH effect, initial concentration of Mn^2+^, temperatures and competing ions were investigated. Adsorption kinetics and isotherms were also examined.

The success of the synthesis of new materials was confirmed by X-ray diffraction (XRD), Scanning Electron Microscopy (SEM) and Energy Dispersive Spectrometry (EDX) analysis that revealed new properties comparative with the basic material, namely brucite. The XRD patterns of LDHs, before and after the Mn^2+^ adsorption, showed that the diffraction intensity of peaks decreases. The XRD pattern presented sharp and symmetrical peaks and some high-angle asymmetrical peaks, demonstrating the formation of a highly crystalline Mg-Al LDH structure. The intensity of peaks observed at 2θ = 42.12° increased after the Mn^2+^ adsorption possibly due to the decreases of the interlayer distance of Mg-Al LDH. SEM images indicate that the LDH particles were uniformly shaped sheets, with most of them being hexagonal. The prepared LDHs exhibited the agglomerates of compact and non-porous structure. EDX revealed that most elements, Mg, Na, O and Al, are present in LDHs. The peak of Ni for Mg-Al-Ni LDH can indicate that the Mn^2+^ was adsorbed (42.64%).

The adsorption equilibrium on prepared LDHs was investigated at different experimental conditions. The distribution of manganese between the liquid and the solid phase in equilibrium was expressed by the Freundlich and Langmuir models. The values of R^2^ show that the Freundlich model offers the best approximation of the retention isotherms of Mn^2+^ for both types of LDH in comparison with the Langmuir model. The adsorption of Mn^2+^ could reach equilibrium quickly at 60 min of shaking time. The pseudo-second-order expression accurately described the Mn^2+^ adsorption kinetics for the prepared LDHs. The adsorption capacity of Mn^2+^ gradually increased with temperature. The Langmuir constant (K_L_) value of Mg-Al LDH (0.9529 ± 0.007 L/mg) was higher than Mg-Al-Ni LDH (0.1819 ± 0.004 L/mg), at 20 °C. Increasing the temperature of the adsorption process increased the adsorption capacity of the Mg-Al LDH. The final removal percentage of Mn^2+^ was higher for Mg-Al LDH (91.85 ± 0.087%) in comparison with the Mg-Al-Ni LDH (35.97 ± 0.093%), at 20 °C. The results indicated that the Mg-Al LDH could be a promising adsorbent that can be used in the removal of other metallic ions from wastewaters.

## Figures and Tables

**Table 1:** Notation of samples (before and after adsorption of Mn^2+^).

Samples	Notation
Mg-Al LDH blank	S1
Mg-Al-Ni LDH blank	S2
Mg-Al LDH—Mn^2+^	S3
Mg-Al-Ni LDH—Mn^2+^	S4

**Table 2:** Values of 2θ and d-spacing (d) of LDHs before and after adsorption of Mn^2+^.

Sample	Phase, System, Lattice Parameters	Pos. (2θ°)	d [Å]	Phase, System, Lattice Parameters	Pos. (2θ°)	d [Å]	Phase, System, Lattice Parameters	Pos. (2θ°)	d [Å]
S1	Mg_4_Al_2_(OH)_14_ 3H_2_O	11.66	7.581	Na_2_CO_3_H_2_O	16.58	5.340	MgAl_2_(OH)_18_	18.36	4.828
	Rhombohedral								
	a (Å) = 3.0380	23.42	3.794	Orthorhombic	21.73	4.083	Monoclinic	20.39	4.350
	b (Å) = 3.08380	39.48	2.280	a (Å) = 10.7000	32.35	2.764	a (Å) = 5.9900	36.84	2.437
	b (Å) = 3.08380	46.97	1.932	b (Å) = 6.4580	32.53	2.749	b (Å) = 7.9700	63.88	1.456
	c (Å) = 22.6200	62.32	1.488	c (Å) = 5.2540	37.94	2.369	c (Å) = 4.3700		
	α (°) = 90			α (°) = 90	42.95	2.104	α (°) = 90		
	β (°) = 90			β (°) = 90	60.87	1.520	β (°) = 91.73		
	γ (°) = 120			γ (°) = 90	62.32	1.488	γ (°) = 90		
S2	NiO	37.23	2.412	Al(OH)_3_	18.41	4.814	NaO_2_	32.42	2.758
	Cubic			Monoclinic			Orthorhombic		
	a (Å) = 4.1800			a (Å) = 8.6410			a (Å) = 4.3350		
	b (Å) = 4.1800			b (Å) = 5.0700			b (Å) = 5.5370		
	c (Å) = 4.2110	43.25	2.089	c (Å) = 9.7190	20.42	4.345	c (Å) = 3.3630	33.39	2.683
	α (°) = 90	62.79	1.478	α (°) = 90	37.23	2.412	α (°) = 90	37.29	2.412
	β (°) = 90	75.28	1.261	β (°) = 94.58	45.17	2.005	β (°) = 90		
	γ (°) = 90	79.25	1.207	γ (°) = 90			γ (°) = 90		
S3	((Mg_6_Al_2_)(OH)	11.42	7.740	Al(OH)_3_	18.35	4.830	MnO	36.96	2.429
	_18_(H_2_O)_4_)_0.375_								
	Rhombohedral			Hexagonal			Cubic		
	a (Å) = 3.0463			a (Å) = 5.0470			a (Å) = 4.2110		
	b (Å) = 3.0463			b (Å) = 5.0470			b (Å) = 4.2110		
	c (Å) = 22.9300	23.15	3.838	c (Å) = 4.7300	18.81	4.713	c (Å) = 4.2110	42.95	2.103
	α (°) = 90	34.91	2.567	α (°) = 90	20.38	4.352	α (°) = 90	46.59	1.947
	β (°) = 90	46.59	1.947	β (°) = 90	42.95	2.103	β (°) = 90	62.32	1.488
	γ (°) = 120	60.89	1.520	γ (°) = 120	62.32	1.488	γ (°) = 90		
S4	Mn_0.2_Ni_7.6_O_8_	37.23	2.413	β-Al(OH)_3_	18.70	4.740			
	Cubic			Monoclinic					
	a (Å) = 8.3495			a (Å) = 5.0100					
	b (Å) = 8.3495			b (Å) = 8.6800					
	c (Å) = 8.3495			c (Å) = 4.7600					
	α (°) = 90	43.25	2.098	α (°) = 90	20.40	4.348			
	β (°) = 90	62.79	1.478	β (°) = 90	40.62	2.219			
	γ (°) = 90	75.28	1.261	γ (°) = 90					

**Table 3:** Summary of Mn^2+^ adsorption isotherm parameters for Mg-Al LDH (sample S1) and Mg-Al-Ni LDH (sample S2), at 10 and 20 °C.

Temperature (°C)	Langmuir			Freundlich		
	K_L_ (L/mg)	a_max_ (mg/kg)	R^2^	K_F_ ((mg/g)/(mg/L)^n^	m	R^2^
Sample S1						
10	0.4061 ± 0.004	1048.320 ± 0.277	0.9759	0.5178 ± 0.237	0.1948 ± 0.004	0.9782
20	0.9529 ± 0.007	1100.607 ± 0.656	0.9586	0.6356 ± 0.311	0.1946 ± 0.004	0.9808
Sample S2						
10	0.2178 ± 0.003	407.537 ± 0.189	0.7282	0.1691 ± 0.614	0.2083 ± 0.005	0.9096
20	0.1819 ± 0.004	436.972 ± 0.260	0.7568	0.1556 ± 0.338	0.2468 ± 0.006	0.9206

**Table 4:** Kinetic parameters and the correlation coefficients of Mn^2+^ adsorption on Mg-Al LDH (sample S1) and Mg-Al-Ni LDH (sample S2), at 10 and 20 °C.

Kinetic Model	Parameters	Sample S1	Sample S2	Sample S1	Sample S2
		10 °C	10 °C	20 °C	20 °C
Experimental	q_e,exp._	483.33	205.50	804.37	350.14
Pseudo-first-order	k_1_, min^−1^	0.1320	0.1550	0.1430	0.1470
	R^2^	0.8832	0.8119	0.8973	0.8580
Pseudo-second-order	q_e,calc._	500.00	314.40	810.00	395.20
	k_2_, g mg^−1^ min^−1^	0.0040	0.0045	0.0017	0.0021
	R^2^	0.9917	0.9671	0.9958	0.9123
